# Pain and Reorganization after Amputation: Is Interoceptive Prediction a Key?

**DOI:** 10.1177/10738584221112591

**Published:** 2022-08-11

**Authors:** Thomas Weiss, Hanna Koehler, Ilona Croy

**Affiliations:** 1 Department of Psychology, Clinical Psychology, Friedrich Schiller University Jena, Jena, Germany; 2 Biomagnetic Center, Jena University Hospital, Jena, Germany

**Keywords:** phantom limb pain, cortical reorganization, interoception, prediction error, predictive coding

## Abstract

There is an ongoing discussion on the relevance of brain reorganization following amputation for phantom limb pain. Recent attempts to provide explanations for seemingly controversial findings—specifically, maladaptive plasticity versus persistent functional representation as a complementary process—acknowledged that reorganization in the primary somatosensory cortex is not sufficient to explain phantom limb pain satisfactorily. Here we provide theoretical considerations that might help integrate the data reviewed and suppose a possible additional driver of the development of phantom limb pain—namely, an error in interoceptive predictions to somatosensory sensations and movements of the missing limb. Finally, we derive empirically testable consequences based on our considerations to guide future research.

## Introduction

**Figure 1.**
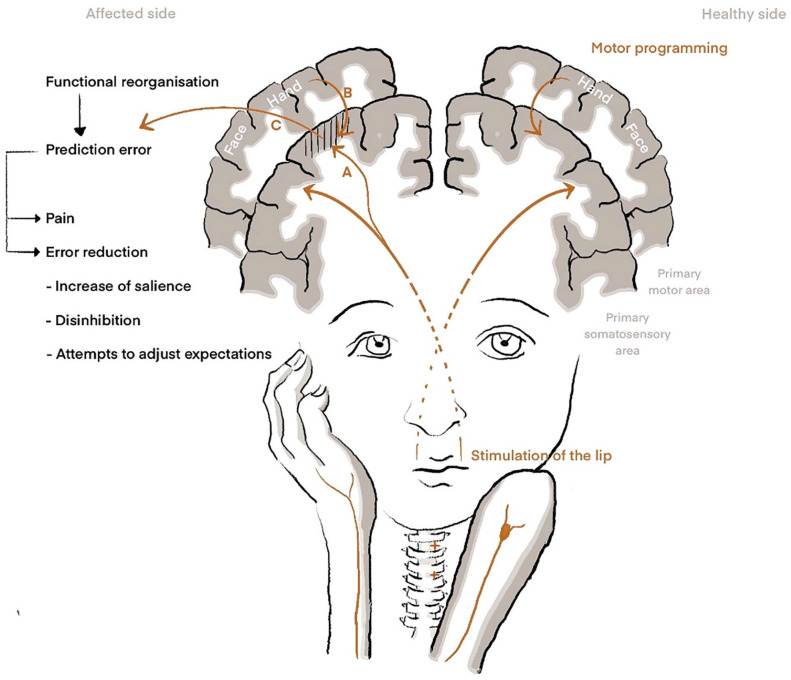
Theoretical models for understanding phantom limb pain. Right: In healthy subjects, the stimulation of the lip results in activation of its representation in the primary somatosensory cortex (S1), while motor programming or motor imagery of the hand leads to activation of sensorimotor representations of the hand. Left: Amputation leads to somatotopic reorganization in the respective contralateral S1 area such that peripheral stimulation of areas in close proximity accesses the S1 area of the amputated limb. Stimulation of the lip hence leads to neural signaling not only within the lip area but also in the area of the amputated hand. (A) The model of maladaptive plasticity indicates that neural activation in the hand area also involves nociceptive neurons and hence directly leads to pain. (B) The model of persistent pain representation suggests that activation of motor programs in the primary motor cortex leads to activation of somatosensory neurons in the respective area. Hence, the imagination of movement of the amputated hand is followed by activation of the reorganized S1 hand area. (C) In the view of predictive coding, we assume that activation of the reorganized S1 hand representation either to peripheral stimulation of neighbor representations that received access or by motor programming leads to a prediction error, as this activation does not match other sensory input or expected sensory consequences for motor control. The prediction error may directly lead to the percept of pain and additionally to attempts for error reduction. Those attempts include 1) the increase of salience processing, which might enhance pain perception, and 2) peripheral and central disinhibition, allowing increased information flow as an attempt to 3) adjust prediction errors.

It has long been disputed why some people suffer from phantom limb pain (PLP; Box 1) after amputation and others do not. In a recent review, Makin and Flor (2020) discuss the relevance of functional brain reorganization in the primary somatosensory cortex (S1). Functional reorganization means that neurons in the somatosensory representation of the amputated body part (e.g., hand) become responsive to similar, usually tactile, input from neighboring representation (e.g., stump or the face). Such reorganization was already observed ~30 y ago in hand amputees (Elbert and others 1994). When those patients were stimulated at a part of their lip, which was ipsilateral to their amputated hand, the central hand region was activated. This was not the case after stimulation on the contralateral healthy side (Elbert and others 1994). Shortly after, Flor and others (1995) extended this finding by demonstrating a strong correlation between the extent of functional reorganization in S1 and the intensity of PLP. Since then, several studies confirmed this association using different types of measurement: electroencephalography (Flor and others 2001; Grüsser and others 2001), magnetoencephalography (Flor and others 1998; Knecht and others 1996), and fMRI (Lotze and others 2001; MacIver and others 2008; Raffin and others 2016). Moreover, the link between S1 reorganization and PLP was affirmed for lower limb amputees (Huse and others 2001; Meyer and others 2012; Zheng and others 2021). Those results formed the basis for the *maladaptive plasticity model* of PLP (Fig. 1), which postulates that input from somatosensory modalities gets access to mechanosensory, nociceptive, and eventually proprioceptive neurons in the neighboring S1 representation (Flor and others 2006; Makin and Flor 2020).

Box 1. **Phantom Limb Sensations, Telescoping, and Phantom Limb Pain**

*Phantom limb sensations*: After losing a limb, nearly all patients report a still present perception of the now missing body part (Giummarra and others 2010; Weiss 2008). Such phantom limb awareness can present as nonpainful sensation, such as tingling, “pins and needles,” or different temperatures and postures (Katz 1992). Sometimes these sensations are so distinct that the person affected can precisely describe them as, for example, wearing a finger ring, wristwatch, shoe, or plaster cast or even as still wearing bandages wrapped around the wound or blood trickling down the limb (Katz and Melzack 1990). Some amputees are able to move the limb, to varying degrees, from turning “only” the wrist to moving the phantom just like the real arm. For instance, a patient reported that he could wiggle each finger or “grab” objects within arm’s reach to the extent of such intuitive movement that he would still grab the receiver of the ringing telephone with his phantom hand (Ramachandran and Blakeslee 1998).

*Telescoping*: Most often, the phantom limb has a shape similar to the limb before amputation. However, over time, the shape of the limb might change. In the phenomenon called “telescoping,” the arm or leg shrinks such that the phantom hand or foot feels closer to the stump or, in the extreme, even diminishes altogether such that it seems to be directly attached to the remaining limb (Katz and Melzack 1990).

*Phantom limb pain (PLP)*: Besides nonpainful phantom limb sensation, 50% to 85% of amputees experience painful phenomena in the deafferented body part (Giummarra and others 2010; Jensen and others 1985; Sherman and Sherman 1983; Stankevicius and others 2021). Such PLP can, like phantom limb sensation, have many qualities. The most frequent pain sensations are stabbing (68%), tingling (60%), electric current (52%), shooting (52%), pulsing (41%), and burning (41%) (Kikkert and others 2018). Notably, the onset of PLP can be immediately after amputation (Weiss and others 2000) but can also occur years after losing the limb (Flor and others 2006). In some patients, the pain decreases over time. However, if the pain lasts >6 mo, the prognosis for positive amendment is poor (Kuffler 2018) such that PLP has a high rate of chronicity and is difficult to treat. Consequently, it is of high importance to understand underlying mechanisms to evolve treatment strategies and improve life quality for patients.

Later observations focusing on the neural representation of the amputated body part itself showed that actual or imagined movements of the missing limb lead to an activation of those S1 areas that previously represented the amputated extremity (Diers and others 2010). Here, Makin demonstrated a direct correlation between the amount of PLP and the activation of the functional representation of the amputated extremity (Makin and others 2013; Makin and others 2015), which led to the *persistent representation model* (Fig. 1). Subsequent studies support the model by replicating the association between activation of the representation of the amputated body part and PLP after movement of the phantom limb (Kikkert and others 2018; Kikkert and others 2016).

At first glance, those two models seem to contradict each other. The maladaptive plasticity model predicts that higher pain levels are associated with greater reorganization as neighboring cortical areas invade the now-deprived area. This remapping after sensory deprivation in amputees coincides with reduced representations in S1. In contrast, the persistent representation model postulates the opposite: thereafter, higher PLP levels are related to maintained representation and therefore are concomitant with increased activity in the sensorimotor cortex of the missing limb. Yet, Makin and Flor (2020) recently addressed these contradictions by presenting their findings as a complementary process in S1. According to them, the differences might result from potential gaps in the layout of the human somatotopy, from the misinterpretation of net activity changes in S1, or, most likely in their view, from the differences in S1 activations resulting from the type of stimulation used in the experiments: passive somatosensory stimulation versus phantom movements. However, the authors acknowledge that 1) both models are not conclusive in explaining the genesis and maintenance of PLP and 2) the functional reorganization in S1 seems not sufficient to explain PLP. Hence, they propose that other brain structures are at least similarly important as S1.

This is needed because maladaptive functional reorganization in S1 cannot sufficiently explain the resulting pain perception. While an amputation leads to a functional reorganization in S1 after amputation in animals (Merzenich and others 1984) and humans (Elbert and others 1994; Flor and others 1995; Makin and others 2013), *it is not convincing that this reorganization results in pain*. First, nociceptive neurons are sparse in S1. Thus, direct S1 stimulations in awake humans do not result in painful sensations (Penfield and Boldrey 1937; Penfield and Rasmussen 1950). Second, the functional significance of nociceptive neurons is relatively low. The direct information flow from spinal cord nociceptive projection neurons to S1 represents ~4%, while the insula receives ~41%, the secondary somatosensory cortex ~29%, and cingulate areas ~24% (Dum and others 2009). The functional role of those neurons, at least in humans, seems to be limited to the onset of nociceptive processing (Hu and others 2015). Third, functional reorganization in S1 was demonstrated through *tactile* stimuli. Such stimuli are processed in the S1 subdivision BA 3b, where the number of nociceptive neurons seems to be neglectable (Dum and others 2009). Therefore, it is quite unlikely that maladaptive functional reorganization in S1 can stimulate a sufficient number of nociceptive neurons in S1 to provide PLP as a consequence. Moreover, the arguments concerning the low number of nociceptive neurons with low functional significance are arguments against the persistent presentation model. These points let us believe that explaining PLP as the result of access of mechanosensitive neurons to nociceptive neurons in S1 is insufficient to explain PLP.

In our view, the *concept of predictive coding* (Friston 2005, 2018; Friston and Dolan 2010) is helpful for the understanding of PLP. Shortly, predictive coding means that a perceptual process involves a continuous interaction between the brain’s model of what to expect and its comparison with actual sensory evidence. Given this, we here present a hypothesis compatible with the data recently reviewed by Makin and Flor (2020). Moreover, it provides an explanation for PLP, including the processing of somatosensory information beside S1. In the following, we give a short overview of predictive coding before explaining a three-step theory of the emergence and maintenance of PLP. Finally, experimentally testable hypotheses are derived to guide future research.

## Predictive Coding as an Explanation for PLP

**Figure 2.**
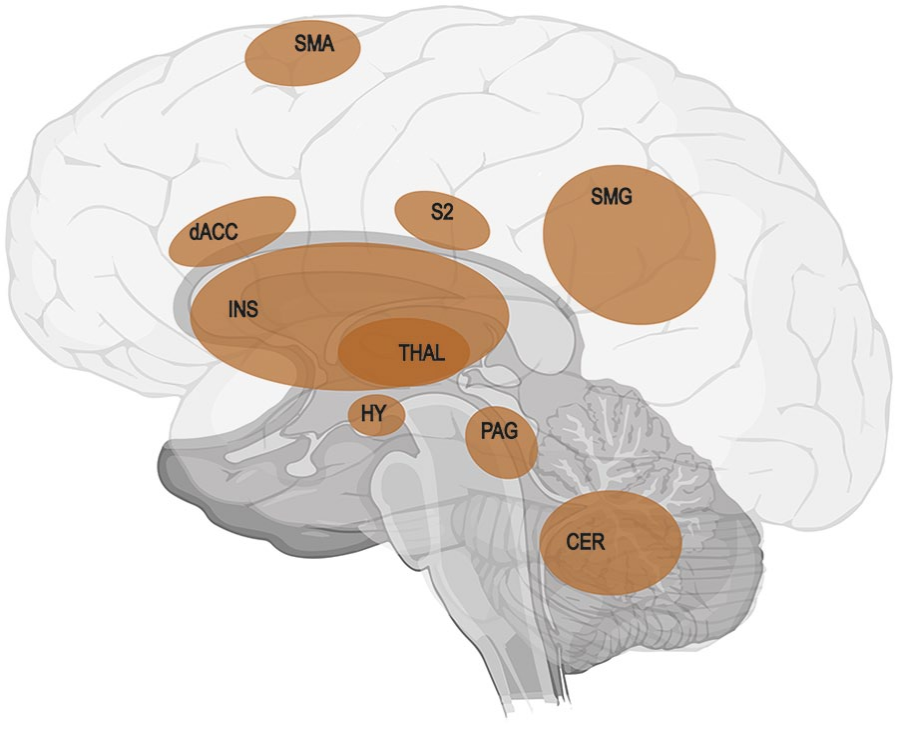
Visualization of the neurologic signature of physical pain identified in a series of four studies by Wager using machine learning analysis for an objective prediction of pain intensity (Wager and others 2013). Red areas show positive predictive values: CER = cerebellum; dACC = dorsal anterior cingulate cortex; HY = hypothalamus; INS = insula; PAG = periaqueductal gray matter; S2 = secondary somatosensory cortex; SMA = supplementary motor area; SMG = supramarginal gyrus; THAL = thalamus. Regions showing negative predictive values were not included in the figure (for these regions, see Wager and others 2013). As the pain signature shares the nodes of the anterior cingulate gyrus and both insulae with the salience network (Ham and others 2013), it enables the initiation of fast and salient processing of pain stimuli.

According to the *concept of predictive coding* (Friston 2005, 2018; Friston and Dolan 2010), perception is not what we sense but the result of the continuous computational outcome between our expectation and the actual sensation or, in other words, from processing the external and internal world within the context of a prior model (Paulus and others 2019). This predictive perceptual processing is hierarchical and bidirectional (Friston 2009). Higher-level models modify the expectations on lower levels, whereas lower levels of the hierarchy provide the input for the higher levels. Predictors (priors) represent the neuronal code of an expected input. Note that such error processing exists already at the lowest levels of processing (e.g., spinal cord processing). An efference copy is an example for this. It will be changed in the course of a learning process. Predictive coding better explains several behavioral findings of somatosensory, interoceptive, and nociceptive processing (Berntson and Khalsa 2021; Chen and others 2021; Paulus and others 2019; Seymour and Mancini 2020; Wiech 2016) than pure bottom-up approaches. In our opinion, predictive coding provides a framework from which a three-step theory of the emergence and maintenance of PLP can be derived: the functional reorganization in S1 leads to a prediction error triggered by peripheral input from neighboring areas and by intentional movements of the amputated limb through M1-S1 feedback. This prediction error then leads to activation of the salience network, which is closely linked to the neurologic signature of pain (Fig. 2) and to peripheral and central disinhibition. Therefore, these mechanisms increase the probability of PLP. In the following, we discuss these individual steps in more detail.

### Step 1: The Triggering of a Prediction Error

A prediction error occurs (Fig. 1) when expectations differ from perceptual input, which might be processed without conscious awareness (Paulus and others 2019).

In hand amputees, for instance, studies demonstrate that the stimulation of the lip results not exclusively in the expected lip representation but in additional activation of central representations of the hand (Elbert and others 1994; Flor and others 1995). Moreover, stimulation at different body locations can result in feelings of the amputated fingers (Knecht and others 1996; Knecht and others 1998), sometimes even with an ordered representation of the fingers at the face (Ramachandran and others 1992). Specifically, Knecht and others (1996, 1998) are among the researchers who investigated this consequently. They found places at which they were able to induce referred sensations to one of the used four modalities in each of their amputees. However, the locations from which they were able to evoke referred sensations were constant for only a short period, at least a day, but differed considerably 4 wk or 1.5 y later. Even more critical in this context, the number of locations correlates with the reported strength of PLP (Knecht and others 1996; Knecht and others 1998). This activation within an unpredicted cortical somatosensory representation should result in a prediction error.

Such prediction errors have two direct consequences, which might be relevant for the development and maintenance of PLP: prediction errors might lead to 1) subjective feelings by prominent, most often aversive, interoceptive symptoms and 2) an obligatory motivation to act to reduce the error and the associated discomfort (Paulus and others 2019). Together, these consequences form the second step of the hypothesis.

### Step 2: Consequence of the Prediction Error—Pain Evoking

**Figure 3.**
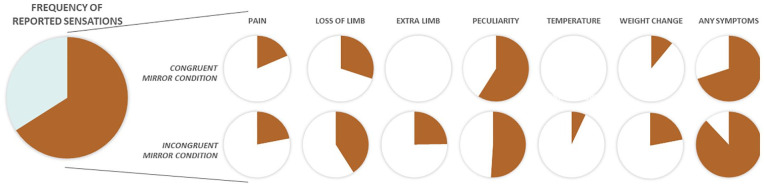
Incidence of physical sensations triggered incongruent visual and motoric information as reported by McCabe and others (2005). The study investigated whether conflicting motor and sensory information processing can lead to pain in healthy volunteers. For this, 41 healthy adults were seated in front of a metal frame with a mirror on the one side and a whiteboard on the other side. They placed one limb on each side of the frame such that one arm and leg were hidden from view. Participants then moved their limbs congruently (e.g., both arms up and down at the same time) or incongruently (e.g., one arm up and the other arm down) while looking at the mirror (visual feedback) or the whiteboard (control). After the movement, subjects described all feelings and changes in either limb: 66% of participants reported symptoms at least in one condition, with most reports in the incongruent mirror condition. Of these participants, most reported symptoms of peculiarity, the perception of the loss of a limb, or having an extra limb and painful sensations. Noteworthy, the participants reported such sensations more often in the incongruent condition than in the congruent condition, which is of high importance with respect to phantom limb pain.

The first consequence directly leads to an aversive sensation (e.g., pain). Motion sickness can serve as a prominent analogy for an aversive sensation or sensory misperception as a consequence of false prediction: motion sickness results from the incongruity of visual versus equilibrium senses—what we see is not what we feel. Moreover, an accompanying prominent symptom in motion sickness, besides nausea and vomiting, is headache (Zhang and others 2016), which suggests a link between prediction error and pain. Concerning the sensorimotor system, another example is the evocation of aversive symptoms such as pain in healthy subjects by provoking prediction errors. McCabe and others (2005) produced various degrees of sensory-motor incongruency using mirrors during congruent versus incongruent movements of the upper and lower extremity in healthy adults. Two-thirds of the participants reported aversive symptoms, including pain (Fig. 3). This pain was described as pins, needles, aching, or shooting pain—descriptors also used by patients with PLP (Montoya and others 1997). Other descriptors were the perceived loss of a limb and the perception of an extra limb, with most sensations being named at the moment of maximal incongruency or maximal prediction error (McCabe and others 2005). However, it should be mentioned that the difference in the percentage of subjects reporting on pain was only 3% in this study.

Another study by Foell and others (2013) investigated a larger number of subjects and controlled for response bias. These authors found a variety of subjective reactions to the congruent and incongruent movement conditions, with perception of a third limb being significantly more intense and frequent in the incongruent condition. Pain was unexpectedly rare in all conditions. One reason for the discrepancy between the results by McCabe and others (2005) and Foell and others (2013) might be that all conditions in the Foell study began with congruent movements making the incongruent condition more intuitive and somehow less unexpected. Congruent movements do not, however, occur in patients with PLP (beside mirror therapy, which might help, as detailed later). Supporting this argument, Daenen and others (2012) compared congruent and incongruent movements in patients with chronic whiplash-associated disorder and healthy controls. All patients cited sensory changes, such as increased pain (60% of patients), tightness (37%), loss of control (34%), and/or dizziness (31%), and several other feelings with lower percentage of occurrence. These reports in patients were independent from test stages. In contrast, 58% of healthy subjects indicated sensory changes, with the highest number of changes during the incongruent mirror stage. This points to possible additional aspects: There might be a threshold of incongruency at which sensory changes including pain might occur, and the system of patients seems to be more vulnerable to incongruencies.

**Figure 4.**
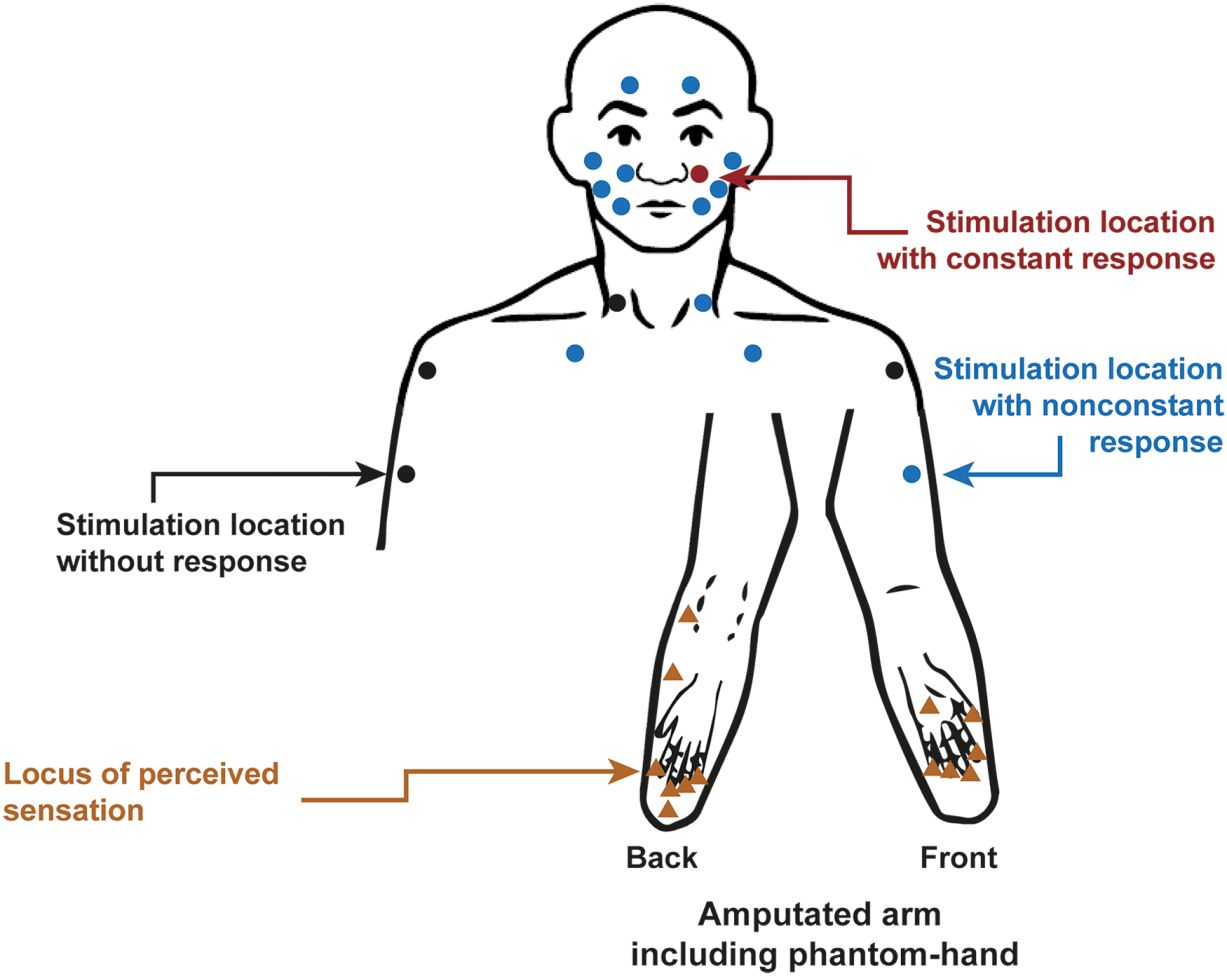
Referred sensations in a patient with amputation of the left forearm and chronic phantom limb pain (PLP) with experience of telescoping. Standardized body sites were touched in the face and the upper trunk with a brush or von Frey hairs with 16 or 512 mN. Each dot represents one stimulation location, each triangle an evoked cramping after stimulation. Although most locations did not constantly evoke PLP (blue), some points never elicited PLP (black), while the stimulation left to the nose (contralateral to the amputation) always evoked cramping in the phantom hand (red). Adapted from Dietrich, Nehrdich, Zimmer, and others (2018).

The link between prediction error and aversive sensation can be transferred to patients with PLP: in healthy individuals, the stimulation of a body part results exclusively in activation of the corresponding cortical representation. That touching the stump in amputees additionally leads to activation of an unrelated area, such as representation of the lip in hand amputees (Dum and others 2009; Flor and others 2001), is highly unexpected and therefore leads to a prediction error and consequently an aversive sensation. In line with this, some subjects regularly report painful perceptions in the phantom in response to nonpainful tactile stimulation of a body part (Dietrich, Nehrdich, Zimmer, and others 2018; Weiss and others 1998) (Figure 4).

So far, prior studies have explained the finding of coactivation in the lip representation by cortical reorganization itself. As the amount of cortical reorganization is linked to the extent of coactivation and therefore to the magnitude of the prediction error, this would explain why the amount of functional reorganization in S1 is directly proportional to PLP (Flor and others 2001). Likewise, the positive effect of therapeutic approaches aiming to reduce cortical reorganization (Dietrich, Nehrdich, Seifert, and others 2018; Dietrich and others 2012; Flor and others 2001; Ortiz-Catalan and others 2016) matches the first consequence, as the retro-reorganization will reduce the maladaptive plasticity and the prediction error.

Two points should be noticed: first, there is a difference between the model of maladaptive plasticity and our proposal. The model of maladaptive plasticity postulates direct activation of nociceptive neurons in S1, while PLP in our hypothesis results from continuous prediction errors in S1. For instance, in mirror therapy, patients place the intact limb in front of a mirror such that the reflection resembles the location of the phantom. The illusion can lead to normalization of the cortical reorganization. Accordingly, Foell and others (2014) found a significant correlation between normalization of lip activity and reduction of PLP after mirror therapy. However, they reported that mirror therapy unexpectedly had no positive effect in patients with telescoping (Box 1). This supports our hypothesis, as the mirror cannot resolve the mismatch and therefore does not reduce the prediction error. Second, the prediction error can lead to aversive symptoms such as pain but not always. This is the case with motion sickness (Zhang and others 2016) as well as in the experiment of McCabe and others (2005), and likewise is valid for PLP, which, although having a lifetime prevalence of 87%, does not affect every patient.

### Step 3a: Consequence of the Prediction Error—Motivation to Reduce the Prediction Error by Increased Salience Processing

The second consequence of the prediction error is the obligatory motivation of the system to reduce the error and the associated discomfort (Paulus and others 2019). This process is expected on each level of the somatosensory system, not only in S1, which gives a hint for additional structures involved in PLP, namely the salience network. The salience network consists of cortical hubs (anterior cingulate cortex, anterior insula) that show common activation patterns whenever internal stimuli of the body or external stimuli become salient (i.e., immediately significant for the organism) (Ham and others 2013; Metereau and Dreher 2013; Seeley 2019). It furthermore represents the neural system for perceiving and responding to homeostatic demands (Seeley 2019). The activation of the salience network is important for action initiation (Lamichhane and Dhamala 2015; Marshall and others 2018). We now assume that a prediction error can activate the salience network and that this process initiates attentional reorientation and conscious action adaptation (Fig. 2).

This assumption is based on the following arguments. The salience network monitors different types of prediction errors, especially in the anterior insula (e.g., Horing and Büchel 2022). If we take into account the function of the salience network, it is not surprising that pain, as a highly negative sensation with behavioral relevance and as a learning-initiating signal, is related to salience network activation. Some authors even argue that the so-called neurologic signature of pain (Wager and others 2013) or neuromatrix of pain (in the sense of Iannetti and Mouraux 2010) is partially identical with the salience network (Legrain and others 2011). This is true not only for acute pain but also for chronic pain in which pain-related stimuli, though not painful per se, can trigger an activation/overactivation of the salience network (Borsook and others 2013). Furthermore, the S1-salience network connectivity is higher in patients with chronic back pain than in healthy controls, and an experimental amplification of pain increases this S1-salience network connectivity (Kim and others 2019). Additionally, a recent meta-analysis (Ferraro and others 2022) found dysregulated anterior insula reactivity as a robust biomarker for chronic pain. Thereby, the anterior insula was identified as the key node of an extended bilateral insula-frontal-cingular network resembling the salience network. In line with those arguments, MacIver and others (2008) reported a higher activation of bilateral insula and anterior cingulate cortex in patients with PLP as compared with healthy controls.

It is essential that the predictive coding framework is a probabilistic theory (Spratling 2017). Accordingly, the salience network response is not uniformly triggered by peripheral input, not even in the same individual. Top-down processes, such as attentional bias, modulate this response. For instance, the emotionality of the context modulates the salience network response: a shift of the focus away from emotional components to nonemotional context leads to a reduction of the response (Iordan and others 2019). In this context, it is interesting that PLP shows a circadian change with maximal pain in the evening—a time of the day where typically fewer competing stimuli occur (Dietrich, Nehrdich, Seifert, and others 2018) to which the focus can be shifted.

### Step 3b: Consequence of the Prediction Error—Motivation to Reduce the Prediction Error by Peripheral and Central Disinhibition

Finally, we assume that the system increases access to peripheral information to solve the prediction error. Increased peripheral information flow is achieved by reducing inhibitory control of the spinal somatosensory processing. Indeed, a common finding after amputation or nerve lesion is the permanent reduction of peripheral inhibition (Costigan and others 2009; Moore and others 2002; Subedi and Grossberg 2011) and central disinhibition (Flor and others 2006). While this reduced inhibition increases the information flow, it strengthens peripheral nociceptive input coming from neuroma, ectopic activation of nociceptive fires, changes in the spinal root ganglia, and so on (for review, see Flor and others 2006), which contributes to the development and maintenance of PLP (Vaso and others 2014).

The decrease of inhibition will not result in the resolution of the prediction error. To illustrate, it does not explain the source of wrong activation of the hand representation while the lip is stimulated mechanically. As such stimulation occurs nearly permanently in everyday life (e.g., by blowing wind at the face), it is plausible that the disinhibition remains permanent, leading to a maintained PLP.

According to the theory of predictive coding, prediction errors lead to a change of prediction. In patients with PLP, a change of expectation might be difficult. For instance, when tactile activation of the lip leads to activation in the deprived cortical hand area, the emerging prediction error should lead to an expectation of a feeling in the missing hand after touching the lip. However, top-down activations (i.e., by motor programming) (Makin and others 2013; Makin and others 2015) will activate the proper representation (meaning the lip) alone and not lead to a feeling in the missing hand, again leading to a prediction error.

## Comparing the Prediction Error Hypothesis with Other Hypotheses on PLP

Not many hypotheses have been presented for the development of PLP so far. One is the hypothesis of “sensorimotor incongruency” by Harris (1999); another is the hypothesis of “stochastic entanglement” by Ortiz-Catalan (2018). We compare these hypotheses with our prediction error hypothesis.

At first sight, sensorimotor incongruency does not differ from our hypothesis. Similar to parts of our argumentation, sensorimotor incongruency postulates that disorganized or inappropriate cortical representation of proprioception may falsely signal incongruency between motor intention and movement. As in our argumentation, this might result in pathologic pain such as PLP. However, there are two significant differences. First, our hypothesis is not restricted to a sensorimotor incongruency but may be related to severe prediction errors within a single system (e.g., motor intention alone). This is important with respect to findings that more severe disturbances in motor control are accompanied by stronger PLP (e.g., Kikkert and others 2017). According to our hypothesis, stronger disturbance in motor control will result in a stronger prediction error within the motor system, as activation of this system results, for instance, in unexpected long reaction times. Second, we believe that the prediction errors, when strong enough, lead to activation of the salience network in its final consequence.

Our hypothesis differs from the hypothesis of stochastic entanglement (Ortiz-Catalan 2018), which proposes a stochastic entanglement of the pain neurosignature or connectome with impaired sensorimotor circuitry. Our hypothesis is clearly different from that of stochastic entanglement, as the latter relates to a triggering of the pain neurosignature by noisy input (or spurious activation), while we see prediction errors within any of the involved systems as the primary source of PLP. At some point, however, the consequence might be similar: activation within the sensorimotor system by noisy input may result in a prediction error.

## Probing the Prediction Error Hypothesis of PLP

The prediction error hypothesis directly leads to four experimentally testable hypotheses.

*Hypothesis 1*: Mirror experiments have shown that triggering a prediction error leads to PLP-like symptoms in healthy individuals (Daenen and others 2012; McCabe and others 2005). We assume that triggering a prediction error leads not only to PLP-like symptoms in healthy subjects but also to a higher probability for PLP in patients after amputation.
*Hypothesis 2*: We hypothesize that triggering a prediction error leads to enhanced activation (BOLD signal increase) in the areas of the salience network and to increased connectivity within the salience network.
*Hypothesis 3*: Activation of the salience network in PLP is stronger when there are no competing other tasks.
*Hypothesis 4*: The occurrence of bursts of PLP is preceded by peripheral stimulation of neighboring areas or by intended movements in patients with PLP and so-called phantom paralysis (Ortiz-Catalan 2018).

While the first two hypotheses can be tested with the experiment of McCabe and others (2005) in an observation study (hypothesis 1) or in an imaging approach (hypothesis 2), the latter two can be tested via experience sampling. Here, patients with noncontinuous PLP record their activities and intended movements preceding each experience of any type of PLP, immediately after the PLP occurrence (or increase in PLP). In addition, the patients are randomly asked to fill out the question of preceding activities and intended movements several times per day independent of the current PLP (control condition). A hint for the possible correctness of hypothesis 4 is an approach of phantom motor execution realized with augmented virtual reality (Ortiz-Catalan and others 2014; Ortiz-Catalan and others 2016). By using this treatment, PLP is reduced when patients gain mobility over the phantom. We assume that by means of phantom motor execution, the prediction error occurring when intending to move a frozen phantom limb is overcome by gaining mobility over the phantom and thereby reducing the prediction error.

## Conclusion

We argue that predictive coding might be helpful to explain both leading models of PLP and additional features of PLP found in amputees. It allows broadening some aspects forwarded by Makin and Flor (2020) concerning the possible substrate contributing to PLP (somatosensory, nociceptive, salience networks). These considerations can be tested, for example, by provoking prediction errors (McCabe and others 2005) or by investigating referred phantom pain (Dietrich, Nehrdich, Zimmer, and others 2018).
